# The Association between Alcohol Consumption and β-Cell Function and Insulin Sensitivity in Korean Population

**DOI:** 10.3390/ijerph13111133

**Published:** 2016-11-14

**Authors:** Min-Gyu Yoo, Hyo-Jin Kim, Han Byul Jang, Hye-Ja Lee, Sang Ick Park

**Affiliations:** Center for Biomedical Sciences, Korea National Institute of Health, Cheongju 28159, Korea; yoomingku@korea.kr (M.-G.Y.); yo2412@korea.kr (H.-J.K.); greatstar@korea.kr (H.B.J.)

**Keywords:** alcohol consumption, insulin sensitivity, β-cell function, hypertriglyceridemia

## Abstract

This cross-sectional study was performed to examine the association between alcohol consumption and insulin secretion and sensitivity using the Korean Genome and Epidemiology Study. Alcohol consumption levels were categorized into four groups: (i) abstainers, (ii) low (<5 g/day), (iii) intermediate (<30 g/day), and (iv) high (≥30 g/day) alcohol consumption. β-cell function and insulin sensitivity were estimated using the insulinogenic index (IGI_60_), and Matsuda insulin sensitivity index (ISI), respectively. IGI_60_ and ISI were dichotomized into high and low groups using median cut-off values and four groups were defined (G-I: high IGI_60_/high ISI; G-II: high IGI_60_/low ISI; G-III: low IGI_60_/high ISI; and G-IV: low IGI_60_/low ISI). Men consumed 26.5 g alcohol per day on average, whereas women only consumed 5.7 g/day, so women were excluded from subsequent analyses due to their low drinking levels. Alcohol consumption was positively associated with high-density lipoprotein (HDL) cholesterol, aspartate aminotransferase (AST), and triglycerides (TG) in men, but was negatively associated with IGI_60_ (*p* < 0.05). TG levels were only increased in individuals with decreased insulin sensitivity (G-II) or decreased β-cell function (G-III) with high alcohol consumption. In addition, alcohol consumption increased HDL cholesterol in the four groups (*p* < 0.001). In subjects with decreased insulin sensitivity (G-II), intermediate and high alcohol consumption increased the risk of high cholesterol and TG. In individuals with decreased β-cell function (G-III), alcohol consumption increased the risk of high TG and high AST levels. High alcohol consumption was significantly associated with reduced insulin secretion. In addition, alcohol consumption was related to some metabolic risk factors depending on insulin secretion or sensitivity.

## 1. Introduction

The prevalence of diabetes mellitus is increasing worldwide as well as in Korea. According to the World Health Organization, diabetes is a major health problem that causes multiple complications, including coronary artery diseases, cerebrovascular diseases, and diabetic neuropathy. Type 2 diabetes mellitus (T2DM) is caused by β-cell dysfunction and the deterioration of glycemic control [1], as well as impaired insulin secretion and sensitivity [2].

Several studies have reported that alcohol consumption is a risk factor for T2DM, possibly due to its effects on insulin sensitivity and secretion [3,4]. Moreover, it has been shown that high alcohol consumption increases the risk of both T2DM and pre-diabetes [5], and in some cases is an independent risk factor for pre-diabetes [6]. In contrast, other studies have shown that light and moderate alcohol consumption protects against T2DM [7,8].

Moderate alcohol consumers with reduced risk of T2DM had increased insulin sensitivity [9,10] through the increase of adiponectin levels. Also, the increase of adiponectin levels after moderate alcohol consumption was associated with improved insulin sensitivity [10]. On the other hand, two studies found that alcohol consumption is associated with β-cell dysfunction [11,12], which is a major risk factor for T2DM. There is no convincing evidence supporting a link between alcohol consumption and β-cell function and insulin sensitivity, both of which are major indicators for T2DM.

According to the Korean National Health and Nutrition Examination Survey in 2013, the alcohol consumption rate in Korea was 75.3% for men compared to 45.7% for women [13]. In addition, recent studies have demonstrated that high alcohol consumption is associated with a higher risk of diabetes in Korean populations [14,15]. It has recently been reported in a Korean cohort study that β-cell function is a more effective predictor than insulin sensitivity in the incidence of T2DM [16]. In the current study, we examined the association between alcohol consumption and β-cell function and insulin sensitivity to determine the possible role of relevant glycemic measures (IGI_60_ for β-cell function and ISI for insulin sensitivity) in the association of alcohol consumption with metabolic risk factors. The link between alcohol consumption and metabolic risk factors was also investigated.

## 2. Materials and Methods

### 2.1. Study Population

Data released from the National Biobank of Korea, the Centers for Disease Control and Prevention (Republic of Korea), and data derived from 10,030 individuals who participated in the Ansung and Ansan cohort study (Korean Genome and Epidemiology Study, KoGES) were used. All of the subjects were adults aged 40–69 years who resided in either rural Ansung or urban Ansan and were enrolled in 2001 and 2002. Detailed information regarding the procedures and design of the Ansung and Ansan study has previously been reported [17]. Individuals with missing data regarding alcohol consumption (*n* = 306) and glucose and insulin levels (*n* = 2125) were excluded. Finally, a total of 7599 participants (3608 men and 3991 women) were included in this study.

All of the participants provided informed written consent. The study protocol was approved by the Korean National Institute of Health’s Institutional Review Board (2016-02-17-PE-A).

### 2.2. Anthropometric and Biochemical Analyses

Body mass index (BMI) was calculated as weight divided by height squared. The fasting plasma concentrations of glucose, total cholesterol, triglycerides (TG), and high-density lipoprotein (HDL) cholesterol were measured using a Hitachi 747 chemistry analyzer (Hitachi Ltd., Tokyo, Japan) following the manufacturer recommendations. Low-density lipoprotein (LDL) cholesterol levels were calculated using the following formula:

(1) $$\mathrm{LDL}\;\mathrm{cholesterol}=\mathrm{Total}\;\mathrm{cholesterol}-\mathrm{HDL}\;\mathrm{cholesterol}-\frac{\mathrm{TG}}{5}$$

The glycated hemoglobin (HbA1c) level was determined by high performance liquid chromatography (Variant II: BioRad Laboratories, Hercules, CA, USA). The plasma insulin concentrations were measured with an immunoradiometric assay kit (INS-IRMA kit; Biosource, Nivelles, Belgium) using a gamma counter system (Packard Instrument Company, Meriden, CT, USA).

Information on physical activity was obtained using a survey with an open question about the hours spent in a typical day at the following levels of physical activity intensity: low intensity, including walking, cleaning, and washing; medium intensity, including fast walking and regular exercise; and high intensity, including athletics, running, and mountain climbing. The amount of physical activity was classified as none or low, medium, and high intensity exercise. Each type of exercise was defined as ≥30 min/day.

### 2.3. Pancreatic β-Cell Function and Insulin Sensitivity

Pancreatic β-cell function and insulin sensitivity were estimated using the oral glucose tolerance tests (OGTT, 75 g). Plasma was obtained 0 min, 60 min, and 120 min after the OGTT to measure plasma glucose and insulin concentrations. Plasma glucose concentrations were measured using the hexokinase method. Plasma insulin concentrations were measured as previously mentioned. The 60-min insulinogenic index (IGI_60_) [18] was calculated using Equation (1):

(1) $$IGI_{60}=\frac{\left({\mathrm{insulin}}_{60}-{\mathrm{insulin}}_{0},\frac{\mu U}{\mathrm{mL}}\right)}{\left({\mathrm{glucose}}_{60}-{\mathrm{glucose}}_{0},\frac{\mathrm{mmol}}{L}\right)}$$

Insulin sensitivity was estimated using the OGTT (75 g) and Matsuda insulin sensitivity index (ISI) after 0 min, 60 min, and 120 min [19]. ISI was calculated using Equation (2):

(2) $$\mathrm{ISI}=\frac{10000}{\sqrt{\left({\mathrm{glucose}}_{0},\frac{\mathrm{mg}}{\mathrm{dL}}\right)\times \left({\mathrm{insulin}}_{0},\frac{\mu U}{\mathrm{mL}}\right)\times \left(\mathrm{mean}\;\mathrm{glucose},\frac{\mathrm{mg}}{\mathrm{dL}}\right)\times \left(\mathrm{mean}\;\mathrm{insulin},\frac{\mu U}{\mathrm{mL}}\right)}}$$

The participants were categorized into four groups (G-I, G-II, G-III, and G-IV) according to their IGI_60_ and ISI scores (G-I: high IGI_60_/high ISI; G-II: high IGI_60_/low ISI; G-III: low IGI_60_/high ISI; and G-IV: low IGI_60_/low ISI). G-I is a reference group. The median value for each index was used to determine the high and low cut-offs [16].

### 2.4. Alcohol Consumption

Alcohol consumption levels were categorized into four groups: participants who did not consume alcohol (abstainers) and those with low (<5 g/day), intermediate (<30 g/day), and high (≥30 g/day) alcohol consumption [20]. Consumed alcohol refers to the collective intake of beer, soju, wine, and liquor in the previous month. The grams of alcohol consumed per day were calculated based on the percentage of alcohol in liquor, the mass of ethanol in 1 liter of liquor, and the glass size (Equation (3)).

(3) $$Alcohol\;\left(g/day\right)=\frac{\mathrm{percentage}\;\mathrm{of}\;\mathrm{alcohol}\;\mathrm{in}\;\mathrm{liquor}\times 0.789\times 10\times \mathrm{glass}\;\mathrm{size}}{1000}$$

### 2.5. Statistical Analysis

Descriptive statistics (mean ± SD or percent) were calculated to present characteristics of the study population according to sex, alcohol consumption, and groups according to IGI_60_ and ISI scores. Student’s *t* tests were used for comparisons between continuous variables, and chi-square tests were used for categorical variables. General linear models (Duncan test as a post-hoc test) were used to compare clinical characteristics and metabolic risk factors by alcohol consumption, the four groups according to IGI_60_ and ISI scores, and alcohol consumption in the four groups according to IGI_60_ and ISI scores. Multiple logistic regression analyses were performed for the associations of the metabolic risk factors with alcohol consumption in the four groups according to IGI_60_ and ISI scores. The association of alcohol consumption with metabolic risk factors was estimated after adjusting for area of residence (Ansung and Ansan), age (continuous), smoking (continuous), and BMI (continuous). Abnormal metabolic risk factors were defined as follows: high total cholesterol (≥200 mg/dL), low HDL cholesterol (<40 mg/dL), high TG (≥150 mg/dL), high LDL cholesterol (≥125 mg/dL), high aspartate aminotransferase (AST) (≥40 IU/L), and high alanine aminotransferase (ALT) (≥40 IU/L). All of the analyses were performed using SAS 9.4 (SAS Institute, Cary, NC, USA). Statistical tests were two-sided, and a *p* value < 0.05 was considered statistically significant.

## 3. Results

General characteristics of the participants in the study are shown in Table 1. The mean alcohol consumption and fasting glucose levels were 26.5 g/day and 88.0 mg/dL for men and 5.7 g/day and 83.8 mg/dL for women, respectively. The percentage of high alcohol consumption for men was 20.4%, whereas that of women was 2.2%. The prevalence of diabetes mellitus was 11.3% for men and 9.0% for women. The geometric means (95% confidence interval, CI) for the IGI_60_ and ISI were 9.9 (9.7–10.1) for men and 8.8 (8.7–9.0) for women. Age, BMI, waist circumference, systolic and diastolic blood pressure, HDL-cholesterol, TG, LDL-cholesterol, AST, and ALT significantly differed between men and women (*p* < 0.05). In the following analyses we excluded women because of the information of minimal alcohol consumption only available in women.

The clinical characteristics of the participants were shown depending on alcohol consumption levels (Table 2). The BMI, waist circumference, systolic/diastolic blood pressure, fasting glucose, HbA1c, total cholesterol, HDL cholesterol, TG, AST, and ALT increased with increase of alcohol consumption (*p* < 0.05), whereas LDL cholesterol and IGI_60_ decreased with increase of alcohol consumption (*p* < 0.05).

Table 3 shows clinical information according to β-cell function and insulin sensitivity. Age, alcohol consumption, BMI, waist circumference, systolic/diastolic blood pressure, fasting glucose, HbA1c, total cholesterol, HDL cholesterol, TG, LDL cholesterol, AST, and ALT significantly differed among the four groups (*p* < 0.01). The frequencies of type 2 diabetes and high levels of alcohol consumption were 1.6% and 16.6%, respectively, in both increased β-cell function and increased insulin sensitivity (G-I); 4.2% and 17.9%, respectively, in only decreased insulin sensitivity (G-II); 23.1% and 22.4%, respectively, in only decreased β-cell function (G-III); and 24.6% and 23.4%, respectively, in both decreased β-cell function and decreased insulin sensitivity (G-IV) (*p* < 0.05).

Table 4 shows the metabolic risk factors characteristics according to alcohol consumption and β-cell function and insulin sensitivity. HDL cholesterol increased with increasing alcohol consumption for all four groups (*p* < 0.05). Moreover, TG increased with increasing alcohol consumption in only decreased insulin sensitivity (G-II) and in only decreased β-cell function (G-III) (*p* <0.05), whereas total cholesterol increased with increasing alcohol consumption in only decreased insulin sensitivity (G-II) (*p* <0.05). LDL cholesterol, AST, and ALT levels were increased in only decreased β-cell function (G-III) (*p* <0.05).

Multivariate logistic regression analyses were performed to determine whether alcohol consumption was associated with metabolic risk factors related to β-cell function and insulin sensitivity (Table 5). In considering participants with only decreased insulin sensitivity (G-II), participants with high alcohol consumption showed an increased risk of high total cholesterol (odds ratio (OR) 1.94; 95% CI, 1.19–3.16) and high TG (OR, 1.99; 95% CI, 1.21–3.27) compared with abstainers (*p* < 0.001). Among participants with only decreased β-cell function (G-III), alcohol consumption increased the risk of high TG (OR, 1.65; 95% CI, 1.02–2.67) and high AST (OR, 3.57; 95% CI, 1.86–6.84) compared with abstainers (*p* < 0.05). In contrast, alcohol consumption was associated with a decreased risk of hypo-HDL cholesterolemia, regardless of IGI_60_ and ISI scores groups (*p* < 0.001).

## 4. Discussion

The current study identified the association between alcohol consumption, IGI_60_ for β-cell function and ISI for insulin sensitivity, and metabolic risk factors, and we also determined the link between alcohol consumption and metabolic risk factors. High alcohol consumption was associated with increased waist circumference, blood pressure, fasting glucose levels, HDL cholesterol, TG, and AST levels, and lower levels of LDL cholesterol and IGI_60_. Participants with only decreased insulin sensitivity had an increased waist circumference, blood pressure, fasting glucose, total cholesterol, TG, and LDL-cholesterol, and decreased HDL-cholesterol levels. In addition, high alcohol consumption was associated with higher levels of total cholesterol and TG in subjects with only decreased insulin sensitivity. Participants with only decreased insulin sensitivity who consumed high levels of alcohol were also more likely to have hypertriglyceridemia compared to abstainers.

Previous studies have shown a U- or J-shaped relationship between alcohol consumption and the incidence of diabetes mellitus. Although light to moderate alcohol consumption was associated with a lower risk of diabetes mellitus [7,8], the pathological mechanisms underlying alcohol consumption in diabetes mellitus are complex. Some studies have suggested that moderate alcohol consumption is associated with improved insulin sensitivity [9,10], whereas a meta-analysis of intervention studies revealed no relationship between moderate alcohol consumption and insulin sensitivity [21]. In addition, studies investigating the effects of alcohol consumption on β-cell function are conflicting. A cross-sectional study reported that insulin secretion was not significantly associated with alcohol consumption [22]; however, other studies have shown that alcohol consumption is associated with β-cell dysfunction [11,12]. Similar results were observed in the current study, in that higher alcohol consumption was associated with lower insulin secretion, but not insulin sensitivity. However, when participants were categorized into four groups according to the extent of β-cell function and insulin sensitivity, alcohol consumption was significantly associated with metabolic risk factors such as total cholesterol and TG.

Insulin resistance and β-cell dysfunction are major risk factors for type II diabetes. Nevertheless, the effects of alcohol consumption on insulin resistance and β-cell dysfunction remain unclear. Several studies have reported that alcohol consumption is associated with lipid profiles, but the effects of alcohol consumption on cholesterol profiles are inconsistent. Total and HDL cholesterol had positive associations with alcohol consumption, whereas LDL cholesterol is not positively associated [23,24,25]. In a study performed in a middle-aged Chinese population, men who consumed ≥30 g alcohol/day had significantly higher total cholesterol levels compared to abstainers, and alcohol consumption was positively associated with HDL cholesterol [24]. A meta-analysis also confirmed that alcohol consumption significantly increased HDL cholesterol levels [25]. These findings are consistent with the positive association between alcohol consumption and total cholesterol and HDL cholesterol in the current study. Also, HDL cholesterol was increased in alcohol consumers from all four groups according to the extent of β-cell function and insulin sensitivity.

There is a positive relationship between TG concentration and alcohol consumption [23,24]. In a cross-sectional study of Korean men, heavy drinkers (≥360 g alcohol/week) exhibited an increased risk of hypertriglyceridemia compared to non-drinkers [23]. In middle-aged Chinese men, alcohol consumption was positively associated with TG [24]. These findings were confirmed by the current study which revealed that TG levels increased with increasing alcohol consumption. Notably, an increased risk of hypertriglyceridemia was observed in participants with only decreased insulin sensitivity or in only decreased β-cell function who consumed high levels of alcohol. It is well established that hypertriglyceridemia is closely linked to insulin resistance. A recent randomized controlled trial reported that reduced TG concentrations were significantly associated with improved insulin sensitivity [26]. However, the association between lipid profiles including TG and both insulin sensitivity and insulin secretion depending on alcohol consumption has not been yet determined.

The liver aminotransferases, AST and ALT, are also associated with an increased risk of T2DM, and several studies have shown that alcohol consumption and insulin resistance are increasingly common causes of metabolic aberrations in the liver [27,28]. Ethanol could work in concert to aggravate cellular injury and fat accumulation [29]. Also, serum ALT activities might be linked with insulin resistance and the biochemical changes occurring during hepatic gluconeogenesis and inflammation (or both) [30,31]. An increase in ALT, but not AST, is associated with a decline in insulin sensitivity [30]. AST and ALT are similar in non-drinkers and individuals who consume <1 drink/day; however, they are increased in individuals who consume 1–2 or >2 drinks/days [32,33]. Also, TG was increased in moderate or high drinkers but not in abstainers or light drinkers, and HDL cholesterol was increased with the increase in drinking [33]. In the current study, AST and ALT levels with TG were higher in the intermediate and high alcohol consumption groups compared to abstainers and the low alcohol consumption group. Also, HbA1c and total and HDL cholesterol were increased in the low alcohol consumption group without the change of AST and ALT levels. In addition, ALT levels were higher in subjects with decreased insulin sensitivity (G-II and G-IV) than with decreased β-cell function (G-I and G-III); AST differed from ALT.

This study has two limitations. First, it has a cross-sectional design; therefore, cause is difficult to determine. However, β-cell function and insulin sensitivity were assessed, and many pre-diabetic participants also had decreased insulin sensitivity. Therefore, this study may represent the effects of alcohol consumption in pre-diabetic subjects. A second limitation of this study is that the type of alcohol was not considered.

## 5. Conclusions

In summary, alcohol consumption was negatively associated with β-cell function, and high alcohol consumption was associated with an increased risk of hypertriglyceridemia in subjects with only decreased insulin sensitivity or only decreased β-cell function. Thus, these data suggest that alcohol consumption may be harmful to individuals at high risk of developing T2DM. Additional studies are required to examine the causal effects of alcohol consumption on the development of T2DM.

## Figures and Tables

**Table 1:** General characteristics of Korean population.

	Total (*n* = 7599)	Men (*n* = 3608)	Women (*n* = 3991)	*p* Value ^6^
Age (years)	52.1 ± 8.9	51.5 ± 8.7	52.6 ± 9.0	<0.001
Alcohol consumption (g/day)	20.8 ± 28.6	26.5 ± 30.9	5.7 ± 11.2	<0.001
Body mass index (kg/m^2^)	24.6 ± 3.1	24.3 ± 2.9	24.8 ± 3.2	<0.001
Waist circumference (cm)	82.6 ± 8.8	83.7 ± 7.6	81.7 ± 9.7	<0.001
Systolic blood pressure (mmHg)	121.6 ± 18.4	122.1 ± 16.9	121.1 ± 19.7	0.025
Diastolic blood pressure (mmHg)	80.4 ± 11.4	82.1 ± 10.9	78.9 ± 11.7	<0.001
Fasting glucose (mg/dL)	85.8 ± 17.1	88.0 ± 18.1	83.8 ± 16.0	<0.001
HbA1c (%)	5.7 ± 0.7	5.8 ± 0.9	5.8 ± 0.9	0.149
Total cholesterol (mg/dL)	191.6 ± 35.6	191.5 ± 35.9	191.6 ± 35.3	0.902
HDL-cholesterol (mg/dL)	44.7 ± 10.1	43.5 ± 10.0	45.8 ± 10.1	<0.001
Triglycerides (mg/dL)	149.5 ± 68.5	159.8 ± 72.5	140.5 ± 63.4	<0.001
LDL-cholesterol (mg/dL)	116.0 ± 32.0	114.8 ± 32.9	117.0 ± 31.1	0.004
AST (IU/L)	29.8 ± 17.8	32.6 ± 20.8	27.4 ± 14.1	<0.001
ALT (IU/L)	27.9 ± 22.3	33.0 ± 25.6	23.3 ± 17.6	<0.001
IGI_60_ **^1^**	5.7 (5.5–5.9)	5.4 (5.1–5.6)	6.0 (5.8–6.3)	<0.001
ISI **^2^**	8.9 (8.8–9.0)	9.9 (9.7–10.1)	8.8 (8.7–9.0)	<0.001
Physical activity **^3^**				
Low (%)	85.5	79.3	91.2	<0.001
Medium (%)	36.7	38.2	35.3	0.009
High (%)	35.7	38.8	33.4	<0.001
Type 2 diabetes (%) **^3^**				<0.001
Pre-diabetes	36.7	49.0	34.4	
Diabetes	7.9	11.3	9.0	
β-cell function and insulin sensitivity (%) **^3,4^**				<0.001
G-I	17.2	18.5	16.3	
G-II	32.8	29.2	35.8	
G-III	29.0	32.3	26.1	
G-IV	21.0	20.0	21.8	
Alcohol consumption (%) **^3,5^**				<0.001
Abstainers	54.0	29.8	75.6	
Low	16.3	14.5	18.1	
Intermediate	18.9	35.3	4.1	
High	10.8	20.4	2.2	

All data except physical activity, type 2 diabetes, β-cell function and insulin sensitivity, alcohol consumption, IGI_60_, and ISI are represented as mean ± standard deviation (SD)s. **^1^** Insulin secretion refers to the insulinogenic index (IGI_60_) and is shown as the geometric mean (95% confidence interval, CI). **^2^** Insulin sensitivity refers to the Matsuda index (ISI) and is shown as the geometric mean (95% CI). **^3^** Physical activity, type 2 diabetes, β-cell function and insulin sensitivity, and alcohol consumption are represented as percentages. **^4^** The medians were used as the cut-offs for separating IGI_60_ and composite ISI values into high and low groups. **^5^** Alcohol consumption was categorized as follows: Abstainers were individuals who did not consume alcohol; low, <5 g/day; intermediate, <30 g/day; and high, ≥30 g/day. **^6^** Student’s *t* tests were used for continuous variables and the chi-square tests were used for categorical variables by sex. HDL, high-density lipoprotein; LDL, low-density lipoprotein; AST, aspartate aminotransferase; ALT, alanine aminotransferase; HbA1c, glycated hemoglobin.

**Table 2:** Clinical characteristics according to alcohol consumption in men.

	Abstainers	Low	Intermediate	High	*p* Value ^3^
*n*	1068	523	1288	729	-
Age (years)	53.5 ± 8.9 ^a^	52.0 ± 8.5 ^b^	51.0 ± 8.7 ^c^	50.3 ± 8.6 ^c^	<0.001
Body mass index (kg/m^2^)	24.1 ± 3.0 ^c^	24.2 ± 2.8 ^b,c^	24.4 ± 2.8 ^a,b^	24.5 ± 2.9 ^a^	0.013
Waist circumference (cm)	83.2 ± 8.0 ^b^	82.8 ± 7.6 ^b^	84.0 ± 7.5 ^a^	84.2 ± 7.5 ^a^	<0.001
Systolic blood pressure (mmHg)	120.9 ± 16.8 ^b^	119.9 ± 17.4 ^b^	122.9 ± 17.1 ^a^	124.3 ± 17.4 ^a^	<0.001
Diastolic blood pressure (mmHg)	80.3 ± 10.5 ^b^	80.3 ± 10.4 ^b^	82.8 ± 10.8 ^a^	83.6 ± 11.6 ^a^	<0.001
Fasting glucose (mg/dL)	87.8 ± 21.1 ^c^	87.3 ± 18.2 ^c^	90.0 ± 22.8 ^b^	94.8 ± 28.9 ^a^	<0.001
HbA1c (%)	5.6 ± 1.0 ^b^	5.7 ± 0.8 ^a,b^	5.8 ± 0.9 ^a^	5.9 ± 1.0 ^a^	0.009
Total cholesterol (mg/dL)	188.6 ± 35.0 ^b^	191.9 ± 34.0 ^a^	192.7 ± 36.5 ^a^	193.1 ± 38.9 ^a^	0.006
HDL-cholesterol (mg/dL)	40.4 ± 8.8 ^d^	42.5 ± 8.8 ^c^	44.7 ± 10.3 ^b^	46.8 ± 10.7 ^a^	<0.001
Triglycerides (mg/dL)	151.0 ± 70.3 ^c^	152.8 ± 69.6 ^c^	164.1 ± 74.0 ^b^	171.4 ± 77.1 ^a^	<0.001
LDL-cholesterol (mg/dL)	117.1 ± 31.3 ^a^	117.6 ± 31.0 ^a^	113.5 ± 33.8 ^b^	110.0 ± 35.4 ^c^	<0.001
AST (IU/L)	30.3 ± 23.0 ^c^	29.1 ± 10.3 ^c^	34.0 ± 25.1 ^b^	36.5 ± 23.8 ^a^	<0.001
ALT (IU/L)	33.0 ± 46.3 ^a,b^	30.5 ± 19.5 ^b^	34.0 ± 30.6 ^a^	35.0 ± 23.8 ^a^	0.048
IGI_60_ **^1^**	6.0 (5.5–6.5) ^a^	6.1 (5.4–6.9) ^a^	5.3 (4.9–5.7) ^a^	4.3 (3.9–4.8) ^a^	<0.001
ISI **^2^**	9.8 (9.5–10.2) ^a^	10.2 (9.7–10.6) ^a^	9.8 (9.5–10.1) ^a^	10.1 (9.7–10.5) ^a^	0.523

All data except IGI_60_ and ISI are represented as mean ± SD. Alcohol consumption was categorized as follows: abstainers, low (<5 g/day), intermediate (<30 g/day), and high (≥30 g/day). **^1^** Insulin secretion refers to IGI_60_ and is shown as the geometric mean (95% CI). **^2^** Insulin sensitivity refers to ISI and is shown as the geometric mean (95% CI). **^3^** General linear models and post-hoc (Duncan) were used to assess variables between different levels of alcohol consumptions. ^a,b,c,d^ Significant differences of means between alcohol consumption levels by Duncan test (a: highest mean; d: lowest mean; a > b > c > d).

**Table 3:** General and clinical characteristics by β-cell function and insulin sensitivity in men.

	G-I	G-II	G-III	G-IV	*p* Value ^3^
*n*	622	1057	1166	723	-
Age (years)	50.8 ± 8.7 ^c^	50.4 ± 8.5 ^c^	53.1 ± 9.0 ^a^	51.9 ± 8.4 ^b^	<0.001
Alcohol consumption (g/day)	23.2 ± 28.4 ^c^	24.9 ± 30.5 ^b,c^	27.9 ± 32.3 ^a,b^	29.3 ± 31.0 ^a^	0.005
Body mass index (kg/m^2^)	23.5 ± 2.5 ^c^	25.4 ± 2.8 ^a^	23.2 ± 2.7 ^d^	25.1 ± 2.8 ^b^	<0.001
Waist circumference (cm)	81.4 ± 6.9 ^b^	86.6 ± 7.3 ^a^	80.9 ± 7.2 ^b^	86.0 ± 7.1 ^a^	<0.001
Systolic blood pressure (mmHg)	118.8 ± 15.6 ^d^	122.7 ± 16.5 ^b^	121.1 ± 17.2 ^c^	126.0 ± 17.2 ^a^	<0.001
Diastolic blood pressure (mmHg)	80.0 ± 10.2 ^c^	83.0 ± 10.9 ^b^	81.0 ± 10.9 ^c^	84.2 ± 10.9 ^a^	<0.001
Fasting glucose (mg/dL)	80.9 ± 7.5 ^d^	86.8 ± 10.3 ^b^	84.6 ± 13.9 ^c^	100.9 ± 29.1 ^a^	<0.001
HbA1c (%)	5.5 ± 0.3 ^c^	5.6 ± 0.4 ^b^	5.6 ± 0.6 ^b^	6.1 ± 1.1 ^a^	<0.001
Total cholesterol (mg/dL)	185.8 ± 33.6 ^b^	196.3 ± 35.5 ^a^	185.8 ± 35.2 ^b^	197.7 ± 37.4 ^a^	<0.001
HDL cholesterol (mg/dL)	44.2 ± 9.8 ^b^	41.3 ± 8.8 ^d^	45.9 ± 10.8 ^a^	42.4 ± 9.3 ^c^	<0.001
Triglycerides (mg/dL)	143.9 ± 62.0 ^b^	176.6 ± 74.8 ^a^	142.1 ± 65.4 ^b^	180.4 ± 77.4 ^a^	<0.001
LDL cholesterol (mg/dL)	112.5 ± 32.1 ^b^	118.5 ± 32.6 ^a^	110.6 ± 32.8 ^b^	117.0 ± 33.8 ^a^	<0.001
AST (IU/L)	29.9 ± 11.0 ^b^	31.2 ± 13.2 ^b^	34.1 ± 24.6 ^a^	34.9 ± 28.2 ^a^	<0.001
ALT (IU/L)	27.9 ± 14.5 ^c^	35.6 ± 22.1 ^b^	30.0 ± 25.1 ^c^	38.4 ± 34.7 ^a^	<0.001
Physical activity ^1^					
Low (%)	84.8	85.1	84.9	87.0	0.237
Medium (%)	38.8	36.0	37.2	35.8	0.285
High (%)	39.1	33.6	39.1	33.9	<0.001
Type 2 diabetes (%) ^1^					<0.001
Prediabetes	27.2	41.5	36.5	45.7	
Diabetes	1.6	4.2	23.1	24.6	
Alcohol consumption (%) ^1,2^					0.024
Abstainers	32.6	30.0	28.3	28.4	
Low	17.1	14.7	14.6	11.8	
Intermediate	33.7	37.5	34.7	36.5	
High	16.6	17.9	22.4	23.4	

All data except physical activity, type 2 diabetes, and alcohol drinking are represented as mean ± SD. ^1^ Physical activity, type 2 diabetes, and alcohol consumption are represented as percentages. ^2^ Alcohol consumption is categorized as follows: Abstainers were individuals who did not consume alcohol. Low, <5 g/day; intermediate, <30 g/day; and high, ≥30 g/day. ^3^ *p* values were determined using general linear models and post-hoc (Duncan) for continuous variables and chi-square tests for categorical variables in the four groups according to β-cell function and insulin sensitivity. ^a,b,c,d^ Significant differences of means between the four groups by Duncan test.

**Table 4:** Metabolic risk factors by alcohol consumption and β-cell function and insulin sensitivity in men.

		Abstainers	Low	Intermediate	High	*p* Value
Total cholesterol (mg/dL)	G-I	183.6 ± 3.2 ^a^	184.9 ± 4.0 ^a^	187.5 ± 4.5 ^a^	189.4 ± 3.9 ^a^	0.435
	G-II	189.7 ± 2.6 ^b^	197.1 ± 3.2 ^a^	199.7 ± 3.8 ^a^	200.4 ± 3.1 ^a^	<0.001
	G-III	183.0 ± 2.2 ^b^	190.6 ± 2.9 ^a^	187.1 ± 3.5 ^a,b^	185.3 ± 2.8 ^a,b^	0.121
	G-IV	197.9 ± 2.9 ^a^	197.2 ± 3.9 ^a^	198.2 ± 5.0 ^a^	199.5 ± 3.7 ^a^	0.962
HDL cholesterol (mg/dL)	G-I	40.7 ± 0.9 ^c^	42.9 ± 1.1 ^b^	46.3 ± 1.3 ^a^	48.0 ± 1.1 ^a^	<0.001
	G-II	38.4 ± 0.6 ^c^	40.9 ± 0.8 ^b^	42.3 ± 0.9 ^b^	44.3 ± 0.8 ^a^	<0.001
	G-III	42.1 ± 0.7 ^d^	44.2 ± 0.9 ^c^	47.3 ± 1.0 ^b^	49.2 ± 0.8 ^a^	<0.001
	G-IV	39.2 ± 0.7 ^b^	40.9 ± 0.9 ^b^	43.7 ± 1.2 ^a^	45.1 ± 0.9 ^a^	<0.001
Triglycerides (mg/dL)	G-I	141.7 ± 6.0 ^a^	137.5 ± 7.3 ^a^	144.9 ± 8.4 ^a^	151.3 ± 7.3 ^a^	0.384
	G-II	158.1 ± 5.6 ^c^	171.8 ± 7.0 ^b,c^	184.9 ± 8.2 ^a,b^	194.6 ± 6.8 ^a^	<0.001
	G-III	130.1 ± 4.1 ^b^	134.7 ± 5.5 ^b^	148.2 ± 6.5 ^a^	153.2 ± 5.3 ^a^	<0.001
	G-IV	181.6 ± 6.3 ^a^	176.7 ± 8.4 ^a^	176.3 ± 10.6 ^a^	187.1 ± 8.0 ^a^	0.563
LDL cholesterol (mg/dL)	G-I	114.6 ± 3.1 ^a^	113.8 ± 3.8 ^a^	111.7 ± 4.4 ^a^	110.4 ± 3.8 ^a^	0.656
	G-II	118.7 ± 2.5 ^a^	120.5 ± 3.1 ^a^	119.4 ± 3.6 ^a^	115.3 ± 3.0 ^a^	0.465
	G-III	114.7 ± 2.0 ^a^	118.0 ± 2.7 ^a^	108.8 ± 3.2 ^b^	104.2 ± 2.6 ^b^	<0.001
	G-IV	120.6 ± 2.7 ^a^	118.6 ± 3.6 ^a^	116.2 ± 4.6 ^a^	115.5 ± 3.5 ^a^	0.449
AST (IU/L)	G-I	27.8 ± 1.0 ^c^	27.3 ± 1.3 ^c^	31.0 ± 1.5 ^b^	34.4 ± 1.3 ^a^	<0.001
	G-II	30.6 ± 1.0 ^a^	30.4 ± 1.2 ^a^	31.3 ± 1.4 ^a^	32.6 ± 1.2 ^a^	0.350
	G-III	29.1 ± 1.5 ^c^	28.4 ± 2.0 ^c^	35.8 ± 2.3 ^b^	40.4 ± 1.9 ^a^	<0.001
	G-IV	30.8 ± 2.2 ^b^	31.8 ± 3.0 ^a,b^	37.0 ± 3.8 ^a,b^	38.4 ± 2.8 ^a^	0.026
ALT (IU/L)	G-I	26.5 ± 1.4 ^b,c^	24.5 ± 1.7 ^c^	28.9 ± 1.9 ^a,b^	32.2 ± 1.7 ^a^	<0.001
	G-II	34.5 ± 1.6 ^a^	36.0 ± 2.1 ^a^	36.2 ± 2.4 ^a^	35.9 ± 2.0 ^a^	0.758
	G-III	28.3 ± 1.6 ^b^	26.5 ± 2.1 ^b^	30.0 ± 2.5 ^a,b^	34.1 ± 2.0 ^a^	0.009
	G-IV	37.9 ± 2.7 ^a^	36.4 ± 3.7 ^a^	39.7 ± 4.7 ^a^	38.9 ± 3.5 ^a^	0.886

All data are represented as mean ± SD. Alcohol consumption was categorized as abstainers, low (<5 g/day), intermediate (<30 g/day), high (≥30 g/day). ^a,b,c,d^ In each row, different letters indicate significant differences of metabolic risk factor means between alcohol consumption levels by Duncan test.

**Table 5:** Metabolic risk factors by alcohol consumption and β-cell function and insulin sensitivity in men.

	G-I				G-II				G-III				G-IV			
	Abstainers	Low	Intermediate	High	Abstainers	Low	Intermediate	High	Abstainers	Low	Intermediate	High	Abstainers	Low	Intermediate	High
High total cholesterol	1	1.08 (0.54–2.16)	0.89 (0.51–1.56)	0.84 (0.44–1.62)	1	1.45 (0.83–2.53)	1.86 ** (1.23–2.82)	1.94 ** (1.19–3.16)	1	0.84 (0.48–1.48)	0.84 (0.54–1.31)	1.14 (0.71–1.84)	1	1.56 (0.73–3.37)	1.11 (0.66–1.88)	1.16 (0.65–2.06)
Low HDL cholesterol	1	0.84 (0.43–1.61)	0.34 *** (0.20–0.60)	0.20 *** (0.10–0.41)	1	0.54 * (0.31–0.94)	0.40 *** (0.27–0.61)	0.30 *** (0.18–0.49)	1	0.58 * (0.34–1.00)	0.35 *** (0.23–0.54)	0.24 *** (0.15–0.40)	1	0.74 (0.36–1.54)	0.35 *** (0.21–0.59)	0.23 *** (0.13–0.42)
High triglycerides	1	0.71 (0.35–1.43)	0.84 (0.49–1.45)	0.94 (0.50–1.75)	1	1.35 (0.78–2.36)	1.83 ** (1.21–2.77)	1.99 ** (1.21–3.27)	1	0.73 (0.43–1.38)	1.12 (0.72–1.75)	1.65 * (1.02–2.67)	1	1.29 (0.60–2.78)	1.01 (0.59–1.72)	1.31 (0.73–2.37)
High LDL cholesterol	1	1.14 (0.56–2.31)	0.67 (0.38–1.20)	0.70 (0.36–1.36)	1	1.65 (0.92–2.95)	1.37 (0.89–2.13)	1.05 (0.63–1.76)	1	0.87 (0.50–1.51)	0.66 (0.43–1.03)	0.72 (0.44–1.17)	1	0.74 (0.34–1.61)	0.80 (0.47–1.36)	0.70 (0.39–1.26)
High AST	1	0.70 (0.18–2.74)	2.31 (0.97–5.53)	2.35 (0.89–6.24)	1	1.76 (0.80–3.90)	1.44 (0.77–2.69)	1.59 (0.78–3.24)	1	0.54 (0.19–1.56)	1.85 (0.97–3.52)	3.57 *** (1.86–6.84)	1	1.15 (0.43–3.10)	1.12 (0.58–2.17)	1.31 (0.64–2.68)
High ALT	1	1.40 (0.52–3.77)	1.24 (0.55–2.83)	1.39 (0.56–3.47)	1	1.44 (0.76–2.73)	1.24 (0.76–2.00)	0.95 (0.54–1.67)	1	0.39 (0.17–0.90)	0.82 (0.48–1.41)	1.23 (0.70–2.15)	1	0.95 (0.43–2.09)	0.57 * (0.33–0.99)	0.66 (0.36–1.20)

Alcohol consumption was categorized into abstainers; low (<5 g/day); intermediate (<30 g/day); high (≥30 g/day); Multivariate logistic regression models were adjusted for area of residence, age, smoking, and body mass index. All data are represented as odds ratios (95% CI); * *p* < 0.05; ** *p* < 0.01; *** *p* < 0.001.
